# From nitrate to NO: potential effects of nitrate-reducing bacteria on systemic health and disease

**DOI:** 10.1186/s40001-023-01413-y

**Published:** 2023-10-11

**Authors:** Hongyu Liu, Yisheng Huang, Mingshu Huang, Min Wang, Yue Ming, Weixing Chen, Yuanxin Chen, Zhengming Tang, Bo Jia

**Affiliations:** Department of Oral Surgery, School of Stomatology, Southern Medical University, Guangzhou, China

**Keywords:** Oral microflora, Esophageal microflora, Gastrointestinal microflora, Nitrate-reducing bacteria, Nitrate reductase, Nitrate, Nitrite, NO

## Abstract

Current research has described improving multisystem disease and organ function through dietary nitrate (DN) supplementation. They have provided some evidence that these floras with nitrate (NO_3_^−^) reductase are mediators of the underlying mechanism. Symbiotic bacteria with nitrate reductase activity (NRA) are found in the human digestive tract, including the mouth, esophagus and gastrointestinal tract (GT). Nitrate in food can be converted to nitrite under the tongue or in the stomach by these symbiotic bacteria. Then, nitrite is transformed to nitric oxide (NO) by non-enzymatic synthesis. NO is currently recognized as a potent bioactive agent with biological activities, such as vasodilation, regulation of cardiomyocyte function, neurotransmission, suppression of platelet agglutination, and prevention of vascular smooth muscle cell proliferation. NO also can be produced through the conventional l-arginine–NO synthase (l-NOS) pathway, whereas endogenous NO production by l-arginine is inhibited under hypoxia–ischemia or disease conditions. In contrast, exogenous NO_3_^−^/NO_2_^−^/NO activity is enhanced and becomes a practical supplemental pathway for NO in the body, playing an essential role in various physiological activities. Moreover, many diseases (such as metabolic or geriatric diseases) are primarily associated with disorders of endogenous NO synthesis, and NO generation from the exogenous NO_3_^−^/NO_2_^−^/NO route can partially alleviate the disease progression. The imbalance of NO in the body may be one of the potential mechanisms of disease development. Therefore, the impact of these floras with nitrate reductase on host systemic health through exogenous NO_3_^−^/NO_2_^−^/NO pathway production of NO or direct regulation of floras ecological balance is essential (e.g., regulation of body homeostasis, amelioration of diseases, etc.). This review summarizes the bacteria with nitrate reductase in humans, emphasizing the relationship between the metabolic processes of this microflora and host systemic health and disease. The potential effects of nitrate reduction bacteria on human health and disease were also highlighted in disease models from different human systems, including digestive, cardiovascular, endocrine, nervous, respiratory, and urinary systems, providing innovative ideas for future disease diagnosis and treatment based on nitrate reduction bacteria.

## Introduction

With the increase in microbiological studies and advances in high-throughput sequencing technology in recent years, several publications on the contribution of microbiota in systemic health and the underlying mechanisms of action have emerged [1–5]. Among them, the bacteria with nitrate reductase are also gaining popularity among researchers [6], which can affect the systemic health and disease of the host by regulating nitrate metabolism [7] (including digestive system [8–11], cardiovascular system [12–14], endocrine system [15–17], nervous system [18–20], respiratory system [21–23] and urinary tract-related diseases [24–26]). There are symbiotic bacteria with NRA (such as micropore bacteria, actinomycetes, *Escherichia coli*, etc.) in the human oral cavity (OC), esophagus and GT, which are closely related to nitrate metabolism [11, 27–30]. Previous studies have shown that these symbiotic bacteria can reduce nitrate to nitrite, increasing nitrite concentration in plasma and saliva. Nitrite is further reduced to NO and exerts its biological activity after being swallowed into the GT [31]. Recent studies have described that DN supplementation can improve cognitive ability [21, 32, 33], skeletal muscle function [34, 35], cardiovascular function [36, 37] and other physiological functions closely related to human health [7, 38–40], and provide some evidence that the bacteria with nitrate reductase are the intermediary of the potential mechanism [41]. One of the mechanisms by which these floras affect human health is their involvement in the production of the signaling molecule NO, which is involved in most physiological activities in the human body (e.g., participation in metabolism and maintenance of cardiovascular homeostasis, dilation of blood vessels, inhibition of atherosclerotic angiopathy, etc.). Therefore, it can be inferred that bacteria with nitrate reductase in the human body are indispensable to human health and disease. The mechanism of its effect on human health deserves further study.

For more than 50 years, DN has been linked to the formation of nitrosamines and the development of cancer [42–46]. As a result, there are strict rules about acceptable levels of nitrate in our diet (e.g., food and drinking water, processed foods and cured meats). It has been observed that the lethal dose of oral nitrate in humans is about 330 mg/kg b.w., and the toxicity of sodium nitrite is about t times that of sodium nitrate. Dietary exposure estimates show that adults consuming 400 g of mixed vegetables does not exceed the daily intake of nitrate, which is within the daily intake range even considering nitrate exposure from other dietary sources. The acceptable daily intake (ADI) of nitrate determined by the former Food Science Council (SCF) was 3.7 mg/kg b.w./day, equivalent to 222 mg of nitrate per day for 60 kg adults and was reconfirmed by the Joint FAO/WHO Expert Committee on Food Additives (JECFA) in 2002. At present, the human intake dose of nitrate and nitrite is within the safe range, far from the toxic dose of nitrate or nitrite [47]. In contrast, in the 1980s–1990s, numerous studies had shown that nitrate could not only biosynthesize in our bodies but also be reduced to NO and other closely related bioactive nitrogen oxides [48–52]. In recent years, it is believed that DN plays a powerful NO-like biological activity in human health [7]. This bioactivity is achieved through a series of reduction reactions. First, nitrite is formed by nitrate-reducing bacteria (NRB) and then transformed into NO and other bioactive nitrogen oxides by non-enzymatic synthesis [53]. With 80% of the body's nitrate coming from dietary leafy green vegetables, studies have shown that naturally occurring nitrate in vegetables is a beneficial active ingredient for systemic health compared to the nitrate added to processed foods and cured meats, and experts generally agree that supplementing nitrate with beetroot juice and other vegetable products may not be harmful [54]. DN is the precursor of signal molecule NO, and DN supplementation can improve NO bioavailability through the NO_3_^−^/NO_2_^−^/NO pathway [55]. NO is a mediator with biological activities, such as regulation of cardiomyocyte function [56], modulation of neurotransmission [57, 58], the principle of platelet function [59], anti-inflammation [60, 61], and prevention of vascular smooth muscle cell proliferation [62, 63]. Various studies demonstrated that supplementation of DN can diastole blood vessels [64], lower blood pressure (BP) [65], and improve oxygen consumption efficiency [66, 67]. One of the mechanisms of the effect of DN on human health is the production of NO through the NO_3_^−^/NO_2_^−^/NO pathway. Among them, NRB, which plays a vital role in nitrate metabolism, are colonized in the digestive tract (including the oral cavity, esophagus, and GT). Most of the nitrate in the human body is reduced in the OC. Therefore, most reports and studies on the effects of NRB in the human body on general health are biased towards oral floras [68, 69]. Considering the critical role of NO in human health, the body’s overall health may be intertwined with the existence of these bacteria. This review summarizes the relationship between the bacteria with nitrate reductase in human floras, the metabolic process, and the general health of the host. Through several disease models of different systems, the importance of bacteria with nitrate reductase in the human body to whole body health was highlighted to study and discover the methods and strategies to treat related diseases by interfering with the growth and metabolism of these NRB in the future.

## Relationship between NRB and their metabolic process and host's general health

### NRB and DN

#### NRB

**Fig. 1:**
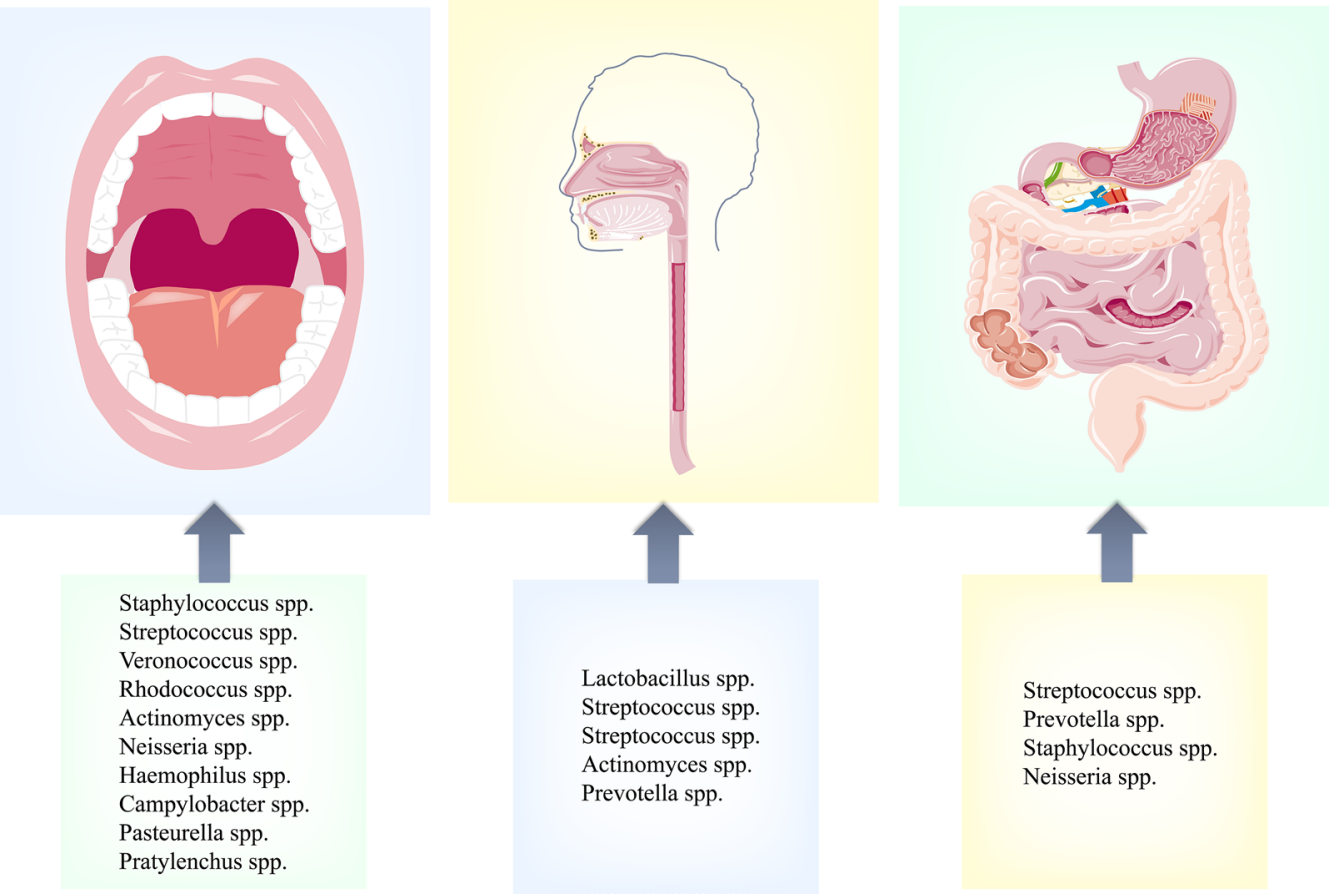
Summary of NRB in the human OC, esophagus, and GT

NRB is a kind of bacteria with NRA. Nitrate is metabolized to nitrite by these symbiotic bacteria and further reduced to NO. This process has become essential to regulate NO homeostasis and signal transduction. There is no nitrate reductase in the human body, but there are symbiotic bacteria with NRA in the human digestive tract (including the OC, esophagus and GT) [11, 29, 31, 37, 69, 70] (Fig. 1). Therefore, NRB play an essential role in the NO_3_^−^/NO_2_^−^/NO pathway in intestinal salivary circulation.

The microbial community of the OC consists of more than 700 prokaryotic taxa and 50–100 billion bacteria (including various NRB) [71–73]. NRB was first isolated from experimental rats in 1997 (including *Staphylococcus* minor, *Staphylococcus* intermedius, *Pasteurella* and *Streptococcus*). In addition, up to 65% of these bacteria are found deep in the posterior tongue [74]. Bacteria with nitrate reductase prefer an anaerobic environment; therefore, they are colonized in the deep fossa of the tongue. The most abundant species include *Prevotella melanogaster*, *Heterotrichia*, *Haemophilus parainfluenzae*, *Neisseria flavus*, *Neisseria fines*, and *Clostridium nucleatum* subsp. nucleatum, *Campylobacter*, *C. labialis*, and *Prevotella intermedia* [75–78]. Recently, some researchers have shown that the NRB in the human mouth mainly includes thick-walled bacteria (*Staphylococcus*, *Streptococcus*, *Veronococcus*), actinomycetes (*Rosella*, actinomycetes), Proteus (*Neisseria*, *Haemophilus*, *campylobacter*, *Pasteurella*), Bacteroides (Proteus) and so on [40]. Regardless of age, the most abundant group of NRB was micropore bacteria, and the level of NRB abundance in the mouth was positively correlated with the amount of DN supplement. The abundance of NRB would increase under a DN load [79]. A comparative analysis of the oral microbiota of vegetarians and omnivores by Hansen and colleagues [80] revealed that a higher proportion of Neisseria and Prevotella was associated with the intake of nitrate-rich vegetables. This suggests that a diet rich in nitrate increases the abundance of NRB and enhances the ability of oral floras to reduce nitrate to nitrite. In addition, the enzymatic activity of NRB varies in a bell-shaped pattern with age (peaking at 30–50 years). It may also vary according to the oral chemical environment (pH, saliva composition, periodontitis), diet type, hygiene practices, and gender [81]. More bacteria with NRA have been discovered in the OC, and the species are more and more abundant, indicating that NRB may play an indispensable role in human health. If NRB is deficient, the NO homeostasis in the body may be broken, and the health of the body will be affected to varying degrees. Tribble et al. [82] have demonstrated through 16S rRNA gene sequencing and analysis that healthy individuals with oral hygiene habits experienced a remarkable reduction in the diversity and abundance of bacteria with nitrate reductase in the mouth after using chlorhexidine mouthwash for a week (twice daily). The decrease in NRB led to a statistically considerable increase in systolic BP, an outcome confirmed in the previous reports [83]. In addition, NRB were also colonized in the oesophagus, which is directly connected to the OC, mainly by *Lactobacillus* spp., *Streptococcus* spp., *Streptococcus* spp., *Actinomyces* spp., and *Prevotella* spp. [84]. Some studies have shown that compared with the normal control group, the concentration of NRB in esophageal effusion samples of patients with non-progressive bulimia is significantly higher, especially in the *Veronococcus* spp., *Lactobacillus* spp. and *Peptococcus* spp. [85].

Gastric intestinal floras have been extensively studied in the last decade, especially the relationship between gastrointestinal floras and human health [86]. Bacteria with NRA (e.g., *Streptococcus* spp., *Prevotella* spp.) are also present in the GT [86–89]. Gastrointestinal NRB may have a role like that of the proven oral NRB involved in nitrate metabolism and further acting on the organism’s health. A nitrate-rich diet has recently been shown to increase the abundance of the faucal phylum bacteroides and reduce the quantity of the thick-walled phylum. This phenotype is associated with lower body weight and mass index [28]. Nevertheless, it has also been noted that 16S amplicon sequencing of faces collected from rats fed with high or low nitrate concentrations for 3 weeks found no differences in microbiome [90]. In addition, Rocha et al. [91] found that under antibiotic-induced bacterial symbiosis disorder, inorganic nitrate supplementation for 1 week could prevent the partial loss of rat faucal microflora, but there was no statistical difference. Does DN affect the metabolism of the gastrointestinal microflora? It has long been confirmed that DN enhances the ability of the GT to resist disease and infection, and antibiotic treatment that inhibits oral NRB increases the sensitivity of gastroenteritis. Nitrate in the diet can reverse the imbalance of gastrointestinal microflora caused by antibiotic therapy and enhance the defense of the GT [92, 93]. It is hypothesized that DN intake may modulate gastrointestinal flora metabolism and promote local redox-reduction interactions, thereby exerting beneficial effects on gastrointestinal flora and health status. Dysregulated NO metabolism is associated with ulcerative colitis (UC) [94]. NO can kill bacteria and regulate gastrointestinal mucosal blood flow (MBF) and mucus production, thus protecting the GT. Therefore, one of the mechanisms by which nitrate affects the GT is the role of NO produced by nitrate metabolism, and NRB takes an irreplaceable part in nitrate metabolism. Most DN is reduced to nitrite in the mouth through the enter salivary cycle. However, some nitrate enter the GT directly through the esophagus. There are more kinds of NRB in the OC, and most (about 80%) of the nitrate is reduced to nitrite in the OC through the intestinal saliva circulation, which is eventually catalyzed to generate other NO-related compounds. Most of them are absorbed by the stomach and upper small intestine before reaching the large intestine, and denitrification rarely occurs in the gastrointestinal tract, especially in the lower intestine [95]. Although it has been reported that long-term exposure to nitrate in food and drinking water is associated with an increased risk of colon cancer, its risk is limited to people with low vitamin C intake and high meat intake, suggesting that its risk may be affected by a combination of food and dietary nitrate [27, 96]. The formation of nitroso compounds (NOC) in the gastrointestinal tract is affected by a variety of environmental factors, including various nitrosation reagents, foods, gastric acid, and intestinal microflora [27]. Nitrate and nitrite in the diet come from vegetables and fruits. These foods contain many nitrosation inhibitors that can prevent cancer development [97]. In addition, European Food Safety Authority (EFSA) also pointed out in its 2008 report that epidemiological studies have not shown that nitrate intake from diet or drinking water is associated with an increased risk of cancer [47]. In summary, dietary intake of natural nitrate is almost impossible to denitrify in the lower intestine and is unlikely to produce carcinogenic NOC; the formation of NOC in the gastrointestinal tract is affected by a variety of environmental factors, and eating fruits and vegetables can inhibit the formation of NOC. Therefore, the current research mainly focuses on the effects of nitrate-reducing bacteria in the oral cavity on systemic health. These results further supported the concept that NRB affects human health through the enter salivary circulating NO_3_^−^/NO_2_^−^/NO pathway. Recently, studies on the effects of NRB on whole-body health have emerged, and the relationship between NRB and organismal fitness has become a focus of research.

#### DN

**Fig. 2:**
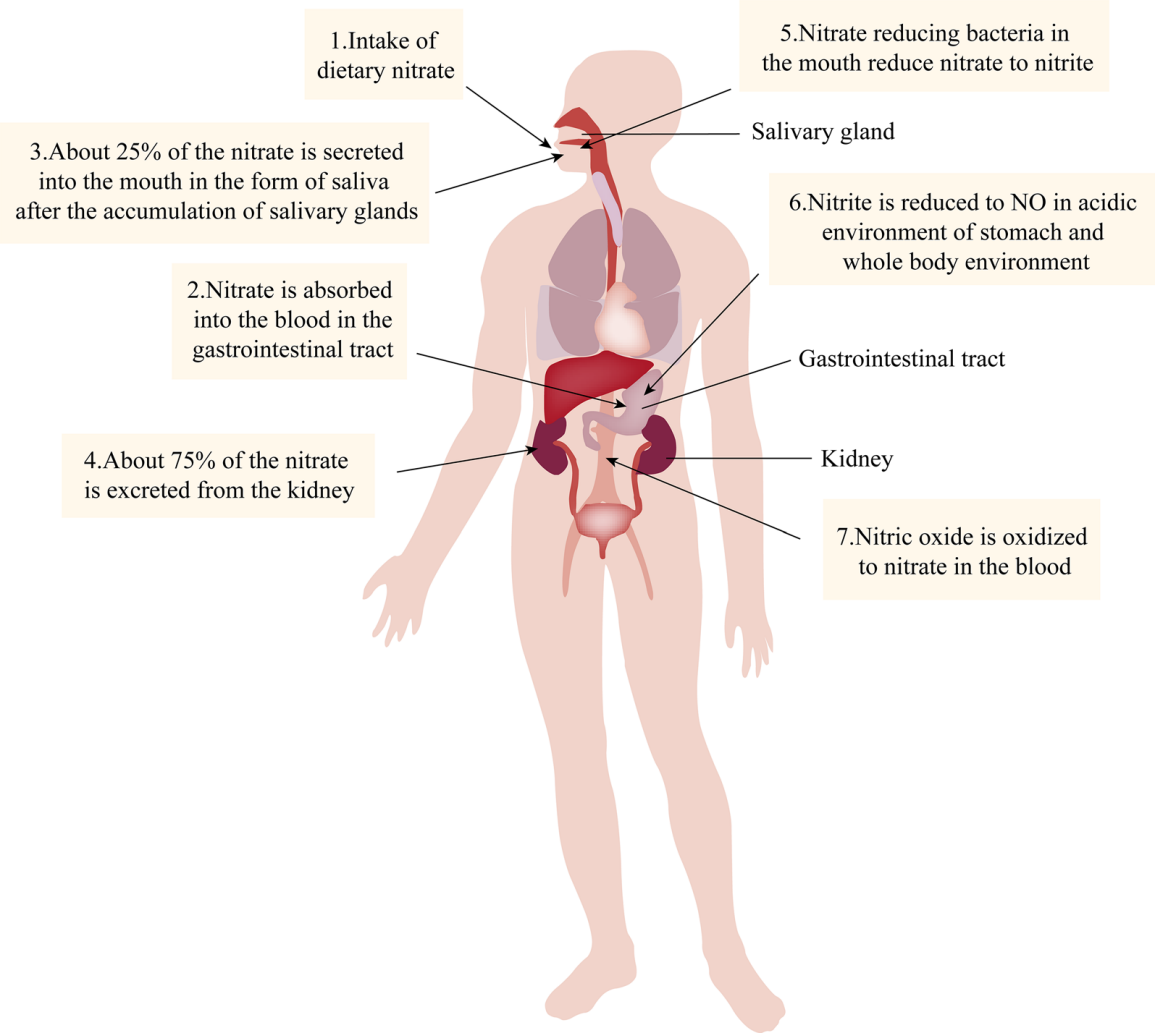
Metabolic process of nitrate in human body

**Fig. 3:**
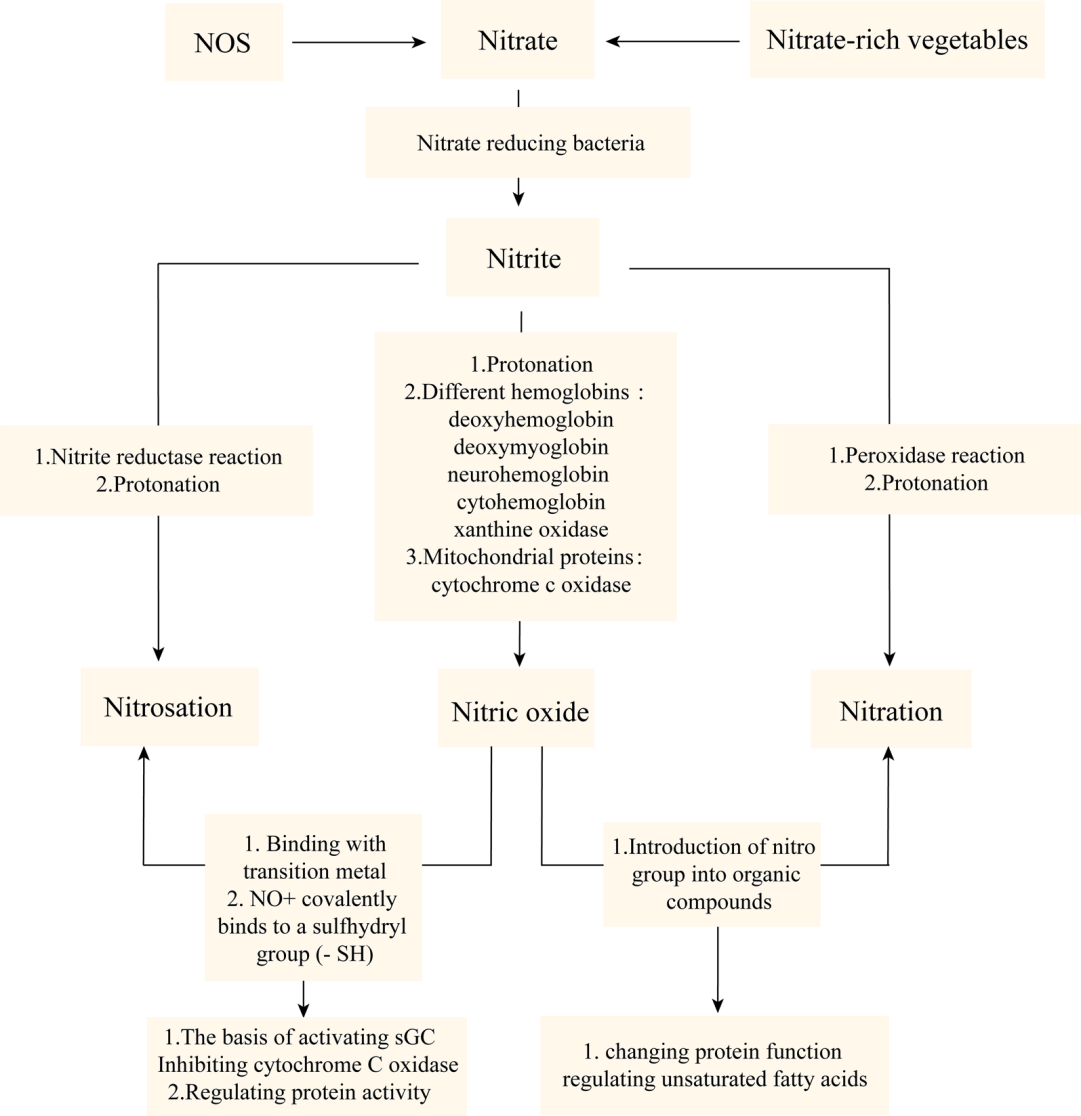
Biochemical processes of nitrate metabolism

**Fig. 4:**
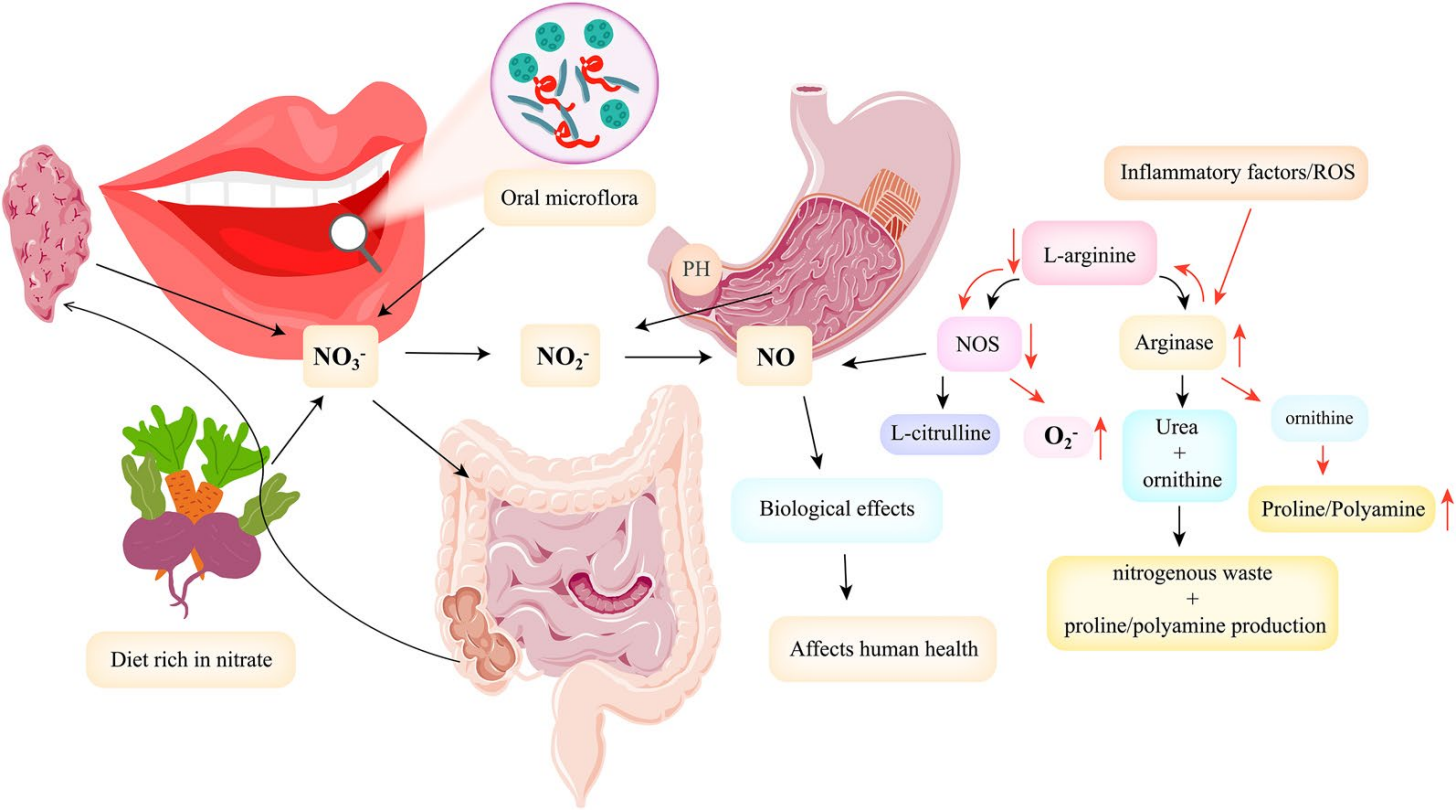
Bacteria with nitrate reductase ultimately reduce nitrate from dietary sources to NO via the enterosalivary cycle; the body can synthesize NO through the l-NOS pathway, but this pathway is inhibited by inflammatory factors or reactive oxygen species stimulation

Nitrate is widely present in water, soil, air, and plants. In addition, nitrate in humans is obtained from two primary sources: dietary intake (exogenous nitrate) and endogenous NO oxidation (endogenous nitrate) [98]. The human body takes nitrate from food, which accounts for about 80–85% of the total nitrate intake [99–101]. DN is primarily found in vegetables, including celery, radish, beet, etc. In addition, nitrate additives contained in meat are also part of the DN source [102]. Most of the DN is absorbed into the blood after entering the GT, and about 75% of the nitrate is excreted into the body in the form of urine through the kidney. About 25% of the remaining nitrate is enriched into the parotid gland with blood circulation and secreted into the mouth in the form of saliva, of which about 20% of the nitrate is reduced to nitrite under the action of NRB in the mouth [103–105]. The remaining nitrate and metabolic nitrite in saliva enter the stomach with swallowing and then enter a new round of the intestinal salivary circulation (80% of nitrite in the human body is produced by the intestinal salivary circulation) (Fig. 2); or NRB can reduce nitrate to nitrite in the acidic environment of the stomach. Then, nitrite is further reduced to biologically active NO and other biologically active nitrogen oxides by acid disproportionation, different hemoglobin (e.g., deoxyhemoglobin, deoxy myoglobin, neurohemoglobin, and cytoglobin), xanthine oxidase, proteins in the mitochondria (cytochrome c oxidase), and nitrite reductase. These nitrogen oxides can be signaled by nitrification, direct NO, and nitridation (Fig. 3) [98, 103, 106]. NO, a metabolite of DN, plays a vital role in protecting the cardiovascular system and gastrointestinal mucosa, regulating cardiomyocyte function and nerve transmission function, and playing a vital role in metabolic diseases. Symbiotic bacteria with nitrate reductase in the human body play an extremely significant role in the NO_3_^−^/NO_2_^−^/NO pathway, which reduces nitrate to nitrite. In addition, NO can be produced through the l-NOS path, a process regulated by NOS and its redox state [107]. Among them, NOS includes three subtypes: neuronal NOS (nNOS), inducible NOS (iNOS) and endothelial NOS (eNOS) [41, 108–110]. However, in the presence of hypoxia, ischemia, disease or ageing, NOS enzyme activity decreases, and endogenous NO production through the l-NOS pathway is reduced. Currently, the NO_3_^−^/NO_2_^−^/NO pathway activity is enhanced to maintain NO homeostasis [111, 112]. NO participates in numerous physiological functions of the human body (such as regulation of cardiomyocyte function, nerve transmission function, platelet function, etc.), so NO homeostasis is closely related to human health (Fig. 4). NO homeostasis is strongly associated with nitrate metabolism. NRB in the OC is essential in the process of nitrate metabolism [113, 114]. In addition, nitrate and nitrite are often used to suppress the development of microorganisms in meat products to prolong their shelf life. Nitrate and nitrite have been considered potential carcinogens for decades and are considered harmful [54, 115]. This stereotype has led to the neglect of the health benefits of nitrate. However, recent studies have failed to prove that nitrate harm humans [116–118]. The World Health Organization (WHO) issued guidelines on nitrate and nitrite in drinking water in 2011, arguing that nitrate are not carcinogens based on laboratory animal and epidemiological studies [119]. In addition, many studies have shown that dietary nitrate has many benefits to human health under the action of NRB (especially oral NRB).

**Fig. 5:**
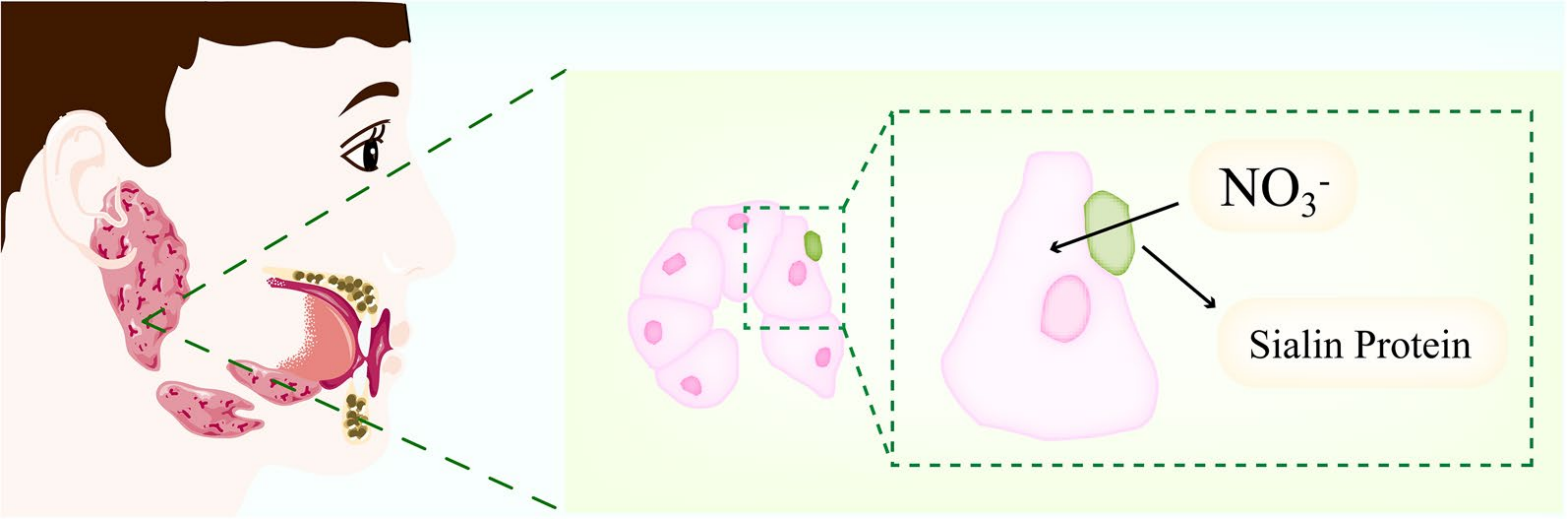
Process of Sialin transporting nitrate in salivary gland cells

In addition, sialin is a membrane protein highly expressed in the parotid gland, a multifunctional anion transporter member of the SLC17 family [120]. Sialin is a salivary gland nitrate transporter with essential physiological effects in modulating NO_3_^−^/NO_2_^−^/NO homeostasis in the body (Fig. 5) [31]. Sialin was distributed in serous vesicle cells and lysosomal basal lateral membrane in the salivary gland [121]. The impaired function of the membrane protein sialin may have deleterious effects on human physiological functions [122]. Qin et al. [121] found proof of the physiological correlation of sialin in transporting circulating NO_3_^−^ into the porcine salivary gland, and inhibition of sialin expression reduced nitrate transport capacity. The discovery that the membrane protein sialin transports circulating nitrate into the parotid gland are critical for future research on the effects of NRB on human disease and health [123, 124].

## Effect of bacteria with nitrate reductase in the human microflora on systemic health

**Fig. 6:**
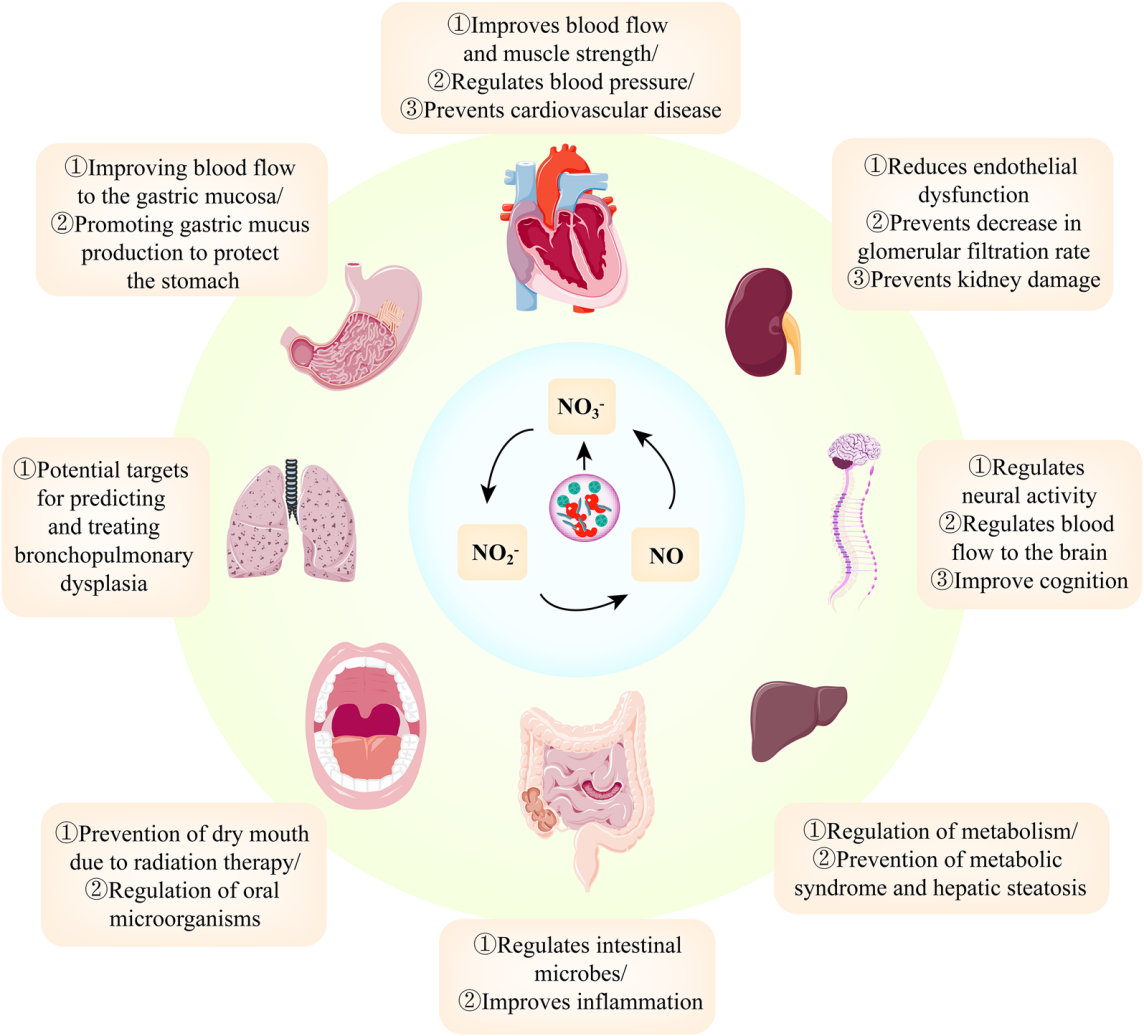
Potential role of NRB in human systemic health and disease through the NO_3_^−^/NO_2_^−^/NO pathway

Many published studies show that DN is beneficial to human health, and NRB and NO play a significant part in the beneficial effects of nitrate. With the increase of age, or in some disease states, the pathway of classical enzymatic reaction to produce NO will be maladjusted. The NO_3_^−^/NO_2_^−^/NO path can compensate for the dysregulation of the classical way. Still, the NO_3_^−^/NO_2_^−^/NO pathway is disrupted when nitrate intake is inadequate, when antibacterial mouthwash/antibiotics are used, and when antacid therapy is used. These two pathways can compensate for each other, but when both systems fail, the NO-based signal will be suppressed entirely, eventually leading to disease. More studies are supporting the benefits of NRB on human whole-body health. From this review, we have discussed the influence of NRB on human systemic health through different disease models (Fig. 6).

### Digestive system

#### Oral-related diseases

There is a lack of effective treatment for salivary gland dysfunction caused by radiotherapy for head and neck tumors (HNT). Feng et al. [125] discovered that nitrate supplementation in the diet effectively prevented radiation-induced parotid hypofunction in miniature pigs. Nitrate is a dose-dependent way to maintain the function of irradiated parotid gland tissue, thus preventing radiation-induced damage to the parotid gland. Nitrate addition to diet was shown to increase the expression of sialin, a nitrate transporter, resulting in a positive feedback loop between nitrate and sialin. It can also stimulate the proliferation of human parotid cells (hPGCs) through the EGFR–AKT–MAPK signaling pathway. Furthermore, it is noteworthy that radiation treatment induces a hypoxic and acidic environment in the salivary gland. Under these circumstances, NO has been synthesized through the NO_3_^−^/NO_2_^−^/NO exogenous pathway, in which NRB is essential in reducing nitrate. Adding nitrate to the diet leads to the production of NO. It decreases hypoxia by inducing a long-term increase in blood flow and increasing glandular micro vascularization, facilitating salivary production by glandular vesicle cells [126]. Furthermore, nitrate-mediated NO generation can enhance the expression of sialin and upregulate the EGFR–AKT–MAPK signaling pathway. This signaling pathway can promote cell proliferation, maintaining cell survival and preventing apoptosis [127, 128]. Therefore, the NO produced by DN through the NO_3_^−^/NO_2_^−^/NO pathway mediated by NRB may be a mechanism for avoiding xerostomia caused by radiotherapy. Recent studies have shown that radiotherapy and chemotherapy in patients with oral cancer (OCC) and oropharyngeal carcinoma (OPC) lead to oral ecological disorders and specific deletion of bacteria regulating the NO_3_^−^/NO_2_^−^/NO pathway in the intestinal salivary circulation [9]. Among them, Neisseria, Haemophilus, Porphyromonas, Clostridium and Cilium disappeared, while Lactobacillus increased, indicating that radiotherapy may lead to NO homeostasis imbalance. This also correlates with the significantly down-regulated oral metabolomic profile of NO-related precursors, regulators, or catalysts (e.g., aspartate, phenylalanine, l-ornithine, l-proline, xanthine, tyrosine, and glycine) in saliva samples from patients after radiotherapy. The decrease in the abundance of these NRB may lead to complications related to NO deficiency, such as xerostomia and local inflammation. Therefore, supplementation of NRB and DN in patients with head and neck radiotherapy and chemotherapy may be a new, safe, and effective method for treating radiation-induced xerostomia. We suggest that future studies explore the likelihood of oral microflora transplantation (OMT) and dietary interventions to reintroduce beneficial microorganisms in the OC of patients after head and neck radiation therapy to improve quality of life.

### Esophagus and GT-related diseases

Considerable evidence suggests that the microbiota has a crucial role in esophageal cancer [129, 130]. Li and his team [11] obtained matched pairs of saliva and esophageal brush samples from 276 subjects who underwent upper gastrointestinal endoscopy, using 16S rRNA analysis and next-generation sequencing technologies to study esophageal microbes. They found that compared with the normal group, the microbial diversity of saliva and cell brush samples decreased with the progression of the disease, and the nitrate reductase function of salivary floras in patients with esophageal squamous cell carcinoma (ESCC) decreased. It was also found that the part of nitrate reductase in matched esophageal brush samples from the same patient had the same change. The microflora in saliva and esophageal cancer cell brush samples are different, but the function of nitrate reductase is weakened, indicating that the bacteria with nitrate reductase can become a sensitive and specific clinical diagnostic marker for ESCC. In addition, it is suggested that NRB play a significant role in regulating the balance of esophageal microorganisms in patients with ESCC, which may be one of the critical mechanisms of the role of esophageal microorganisms in oesophagal cancer.

In addition to the esophagus, NRB can also be found in the stomach and may be closely associated with human health [86, 89, 131, 132]. A research team has, for the first time, published a report on the composition of gastric microflora during the development of gastric cancer. They found NRB (including *Neisseria*, *Clostridium*, and staphylococci) in the stomach and believed these bacteria were potential candidates associated with gastric cancer. However, the actual role of these bacteria in the development of gastric cancer has not been evaluated [86]. In addition, there is preliminary evidence that DN can protect the stomach by inducing gastric mucosal vasodilation, improving gastric mucosal blood flow, and promoting gastric mucus production. Recent studies suggest that these observations may be caused by NRB reducing nitrate through the NO_3_^−^/NO_2_^−^/NO pathway to produce NO in the human stomach [93, 133, 134]. Therefore, the specific effect of NRB on the stomach is worthy of our in-depth study.

DN can alter the gut microbial ecology system [75, 135, 136]. Hu and his colleagues [137] have demonstrated that DN supplements prevent colitis from upregulating carcinogenic pathways implicated in colorectal cancer development (e.g., activation of p53), indicating that nitrate may regulate inflammation by reshaping gut microbes. Previous studies have found reduced bacterial diversity and increased bacterial instability in patients with IBD compared to healthy individuals [138–140], confirming the changes in the microbiota of patients with IBD. However, the pathogenesis of IBD is still not fully understood, and a primary cause may be linked to the unbalance of intestinal bacteria. To investigate the mechanistic details leading to the dysbiosis of the intestinal bacteria in IBD, investigators assessed the role of nitrate in a mouse model of dextran sulfate sodium salt (DSS)-induced colitis [137]. The results revealed that nitrate addition to the diet maintained colonic consistency, improved colonic length, increased microvascular density, modulated serum Th cells, and decreased apoptosis rate in colonic epithelial cells, indicating that nitrate supplementation could reduce experimental colitis in mice [28, 137]. This suggests that nitrate can significantly ameliorate DSS-induced colitis by reducing the inflammatory response, decreasing apoptosis of colitis cells, improving intestinal blood flow, and activating the NO_3_^−^/NO_2_^−^/NO pathway to regulate intestinal floras [28, 141, 142]. Therefore, one of the pathogenic mechanisms of IBD may be that l-arginine-dependent endogenous NO synthesis is inhibited under inflammation, while exogenous dietary nitrate activates the NO_3_^−^/NO_2_^−^/NO pathway under the action of NRB to restore the balance of intestinal floras.

### Cardiovascular system

#### Hypertension and pulmonary hypertension

Although we have improved the way we diagnose and image cardiovascular diseases (CD) at an early stage, with many new drugs approved for the treatment of CVD (such as hypertension, etc.), hypertension still puts us at risk of heart disease and stroke, which is the leading cause of death around the world [143]. Although the pathophysiological aspects of hypertension have been intensively studied in these decades, its incidence and prevalence have not decreased significantly. It is assessed that even with active drug therapy for hypertension, only approximately 50% of patients have their BP under control [25, 143]. At present, looking for new targets to prevent and treat hypertension is still the direction of our efforts. In 2006, the first study showed that inorganic nitrate reduced diastolic BP (3.7 mmHg) in healthy subjects 3 days after taking sodium nitrate (0.1 mmol/kg) [144]. Nitrate was later found to affect systolic blood pressure in a similar group of subjects [145–147]. A subsequent study validated previous claims that healthy volunteers reduced systolic and diastolic BP by 10.4 mmHg and 8 mmHg, respectively, after taking 500 ml of beetroot juice [147]. Interest in the effects of nitrate on cardiovascular function has been raised by these studies showing the potential of DN to lower BP. In recent years, it has been indicated that imbalances in the oral microbial community can negatively impact cardiovascular and metabolic health. Nitrate can produce NO in the human gastrointestinal tract through the NO_3_^−^/NO_2_^−^/NO pathway mediated by NRB, which may be one of the essential mechanisms of nitrate lowering BP in patients [12–14, 41, 148]. Recently, a review has elucidated the role of NO in the fight against CD, including hypertension, among others [149]. Evidence has shown that hypertension can be offset by drugs that improve NO signaling or restore NO bioavailability (for example, angiotensin-converting enzyme (ACE) inhibitors promote elevated levels of bradykinin, which activates the bradykinin B2 receptor in endothelial cells and eNOS, which increases NO production) [108]. Carlström et al. [150] further described the effects of dietary nitrate on various organ systems through the NO_3_^−^/NO_2_^−^/NO pathway under the action of NRB and discussed the potential mechanism of dietary inorganic nitrate reducing BP.

Small reductions in systolic BP in groups of people can remarkably decrease the risk of hypertension and mortality from CD (e.g., stroke). Therefore, exploring the effect of NRB on systemic blood pressure is significant. Evidence from studies has revealed that untreated hypercholesterolemic patients who consumed beetroot juice rich in nitrate for 6 weeks increased the abundance of NRB in the microflora of saliva while improving brachial flow-mediated dilation and reducing platelet monocyte aggregation [151]. More recently, Vanhatalo and his colleagues [14] showed in a clinical study that supplementation with nitrate-rich beet juice for 10 days similarly increased the abundance of NRB (*Rhodobacter* spp. and *Neisseria* spp.) in the salivary microbiota in both older (70–79 years) and younger (18–22 years) healthy subjects. They also found that acute (10 days) nitrate supplementation only reduced BP in healthy older adults, not in healthy younger adults. The current research results show that the oral microbial community is plastic and changes with the change of dietary inorganic nitrate intake, and the diet-induced changes in the oral microbial community are related to NO homeostasis and vascular health index. It has been shown that antimicrobial mouthwash reduces the number of NRB in the OC and can impair the antihypertensive and vasoprotective effects of l-arginine [152]. To explore the potential relationship between NRB and systemic BP, Petersson et al. [153] gave rats antibacterial mouthwash twice a day and supplemented nitrate drinking water simultaneously. They found that the simultaneous use of mouthwash significantly reduced the number of oral NRB compared with rats fed only nitrate drinking water, offsetting the drop in systemic BP caused by nitrate supplementation. More recently, data from various sets of animal models and humans have also revealed that antibacterial mouthwash can decrease the concentration of nitrite in the mouth and plasma while increasing BP by 2–3 mmHg [152–156]. Senkus et al. [83] performed an in-depth dissection of eight published studies between 2009 and 2016 (including five human crossover studies and three animal control studies). The data indicated that applying antibacterial mouthwash negatively affects saliva and plasma nitrate/nitrite concentrations, accompanied by increased BP. A review has elaborated that the disturbance of NO homeostasis by antibacterial mouthwash may cause an increased risk of cardiovascular mortality [53]. These findings demonstrated that DN supplementation increased the abundance of NRB, and that salivary nitrate can modulate cardiovascular function through the bioactivation of oral commensal bacteria. In contrast, overuse of antibacterial mouthwash may diminish the biological activity of DN. Goh et al. [78] pointed out that oral NRB can benefit the modulation of BP. Exploiting the NRA of specific symbiotic bacteria to make the NO_3_^−^/NO_2_^−^/NO pathway a potential system for maintaining NO bioavailability requires far-reaching and truly transformative research. Future longitudinal studies will strengthen the evaluation of the relationship between NRB exposure and hypertension, predict biomarkers of cardiometabolic risk and clinical disease progression, achieve early prevention of disease risk, and will provide information for the evolution of further intervention research manipulating oral NRB.

To explore the deeper mechanisms of the NO_3_^−^/NO_2_^−^/NO pathway to lower BP, Guimarães et al. [18] administered DN supplementation to angiotensin II (Ang II)-induced hypertensive rats and found that inorganic nitrate treatment not only reduced oxidative stress by promoting the NO_3_^−^/NO_2_^−^/NO pathway but also resulted in the suppression of sympathetic nerve activity (SNA) in this animal model or even normalized it, ultimately reducing BP in this animal model. They found that enhanced SNA boosts the progression of the disease, including hypertension, and raises the risk of adverse complications. A novel strategy to inhibit SNA may be of immense value in preventing or treating hypertension. Promoting the NO_3_^−^/NO_2_^−^/NO pathway through DN supplementation is a new nutritional and pharmacological approach to SNA inhibition in which NRB play an irreplaceable key role. However, this trial did not clarify that acute and chronic supplementation with inorganic nitrate can suppress SNA in hypertensive patients.

Recent studies have shown that *S*-nitrosation is impaired in hypertension, and increasing this modification may be an effective antihypertensive strategy. It is worth noting that NO plays a vital role in activating guanylate cyclase (GC), especially through the *S*-nitrosation of proteins. Nitrosylation affects G protein-coupled receptor (GPCR)-mediated signaling and can alter the affinity of angiotensin II for angiotensin II type 1 receptor and β-blocker transport [108]. These evidence suggests that this NO-mediated retro-translational modification may be closely related to vascular regulation and that NRB is a crucial contributor to NO production. Thus, NRB may regulate protein *S*-nitrosation through the NO_3_^−^/NO_2_^−^/NO pathway and thus effectively counteract hypertension.

In addition, it has been shown that when brassica vegetables rich in thioglucosides are consumed dietary (e.g., cauliflower and broccoli), mustard enzymes can convert thioglucosides to thiocyanate and increase serum thiocyanate levels [108]. Eating vegetables rich in nitrate can lower BP, but this effect will be eliminated when eating vegetables rich in nitrate and thiocyanate simultaneously. To investigate how this thiocyanate affects the antihypertensive effect of nitrate, Dewhurst-Trigg and his colleagues [157] found that consumption of thiocyanate-rich vegetables did not impact salivary nitrate intake but may inhibit the activity and metabolism of NRB, thereby affecting the capacity for nitrate conversion to nitrite in the OC. In addition, smoking increases the cycle level of thiocyanate, because cyanide in cigarette smoke is easily converted to thiocyanate through a desulphurization reaction catalysed by thiosulfate and 3-mercaptopyruvate. The study found that compared with non-smokers, smokers increased and decreased the concentration of nitrate in plasma and saliva while higher levels of thiocyanate in plasma and saliva. These phenomena are related to eliminating antihypertensive effects after smoking dietary nitrate in smokers. Both dietary intakes of thiocyanate-rich vegetables and smoking impair nitrate metabolism and the antihypertensive effect on this anion. It was recently demonstrated that smokers significantly impaired the conversion from nitrate to nitrite in the OC, suggesting that altered activity of oral NRB may be an essential pathophysiological mechanism.

Chronic gestational hypertension is significantly associated with poor outcomes in pregnancy, raising the risk of preeclampsia [158, 159], fetal growth restriction [160], and preterm birth. Dietary supplementation with nitrate has been shown to restore NO balance in the body, improve endothelial dysfunction, and lower BP. A clinical study has demonstrated a potential effect of DN (beet juice) supplementation on BP in hypertensive pregnant women [161]. The NO_3_^−^/NO_2_^−^/NO pathway process primarily involves the activity of bacterial nitrate reductase. It is hypothesized that variations in the effects of nitrate supplementation are associated with differences in individual microbiomes. Further studies must confirm the relationship between chronic gestational hypertension and NRB. Future trials should explore and assess the beneficial influence of probiotic supplementation on the prognosis of pregnant women with hypertension. Probiotic interventions and supplementation with DN may suggest a safe and effective way to treat hypertensive disorders of pregnancy.

Moreover, pulmonary arterial hypertension (PAH) is a vascular disease in which mechanical obstruction increases in mean pulmonary arterial pressure [162]. Endothelial dysfunction, inflammatory and immune responses, and abnormal extracellular matrix function play a key role in PAH. PAH has vascular endothelial dysfunction and low NO bioavailability. Studies have shown that low-dose inhalation of NO (lasting for 4 h at 20 ppm concentration and then for 20 h at 6 ppm concentration) can improve oxygenation in PAH newborns without affecting the whole body and reduce systolic BP in nine PAH newborns [163, 164]. Subsequently, Roberts and his team [165] confirmed that inhaled NO increased systemic oxygen levels through a multicenter randomized controlled study of full-term and near-term infants with PAH. In addition to in vitro NO supplementation, restoration of NO bioavailability through dietary supplementation with NRB may be a dietary therapy for PAH.

As discussed earlier, the NRB-mediated NO_3_^−^/NO_2_^−^/NO pathway performs a vital role in preventing the progression of CD, for example, by reducing arterial stiffness, improving endothelial function, and reducing the risk of CD [12]. In addition, a recent study by feeding nitrate-added drinking water to mice with chronic ischemia in the hind limbs for 2 weeks found increased mobilization of CD34(+)/Flk-1(+) cells and migration of bone-marrow-derived (BMD)CD31(+)/CD45(−) cells to the site of ischemia, correlating with enhanced revascularization [166, 167]. These BMD endothelial progenitor cells (EPC) are activated in response to vascular stress and injury and are engaged in angiogenesis and restoration. The regenerative effect of DN can be abrogated using an antibacterial mouthwash, suggesting the importance of NRB in the NO_3_^−^/NO_2_^−^/NO pathway. Subsequently, the same acute mobilization of EPC was observed in the same studies after nitrate supplementation in humans [167–169]. These findings reveal that NRB may protect vascular function by mediating the NO_3_^−^/NO_2_^−^/NO pathway to affect the release and migration of various BMD cells.

However, due to the short duration and small sample size of the completed studies, the proof of the long-term effects of DN on BP in patients at increased cardiovascular risk is still not conclusive at this point. Therefore, future researchers need to use more accurate methods to evaluate the benefits and potential mechanisms of NRB in CD, such as hypertension, in clinical trials with a larger scale (> 300 participants) and longer DN supplementation time (> 12 months). The NO_3_^−^/NO_2_^−^/NO pathway determines NO homeostasis in cardiovascular health and disease. NRB plays a vital part in this pathway, providing a new target for the treatment of hypertension. We can modify CD patients’ prognosis through DN and probiotic supplementation. The findings of Goh et al. [78] support the role of oral NRB having a beneficial effect on BP modulation and insulin resistance (IR), which is one of the first studies to directly test the priori established hypothesis that oral NRB influences cardiometabolic outcomes. Future longitudinal studies will strengthen the estimation of the potential of NRB as biomarkers for predicting cardiometabolic risk and clinical disease progression and provide information for the evolution of future intervention studies that may manipulate oral nitrate-reducing capacity.

### Heart disease

According to the most recent statistics on factors associated with heart disease reported annually by the American Heart Organs Association and the National Institutes of Health, diet is one-factor affecting heart health [170]. Nitrate is a potential dietary supplement for lowering BP. NRB acts as a key “driver” for lowering BP in patients through the NO_3_^−^/NO_2_^−^/NO pathway, with few studies investigating its effects on cardiac function. Under normal conditions, the decrease in BP due to vasodilation usually causes an increase in pressure reflex activity and heart rate, but with DN supplementation, only a drop in BP and no change in heart rate was observed [171, 172]. There are two explanations for this result: first, the effect of NO_3_^−^/NO_2_^−^/NO pathway mediated by NRB on BP is not enough to cause an increase in baroreflex activity and heart rate; second, NRB may have a direct or indirect inhibitory effect on baroreflex. Beetroot juice can be used as a natural nitrate supplement to increase the concentration of nitrite in plasma under the action of NRB [173]. In isolated and perfused Langendorff rat heart models, it has been shown that the increase of plasma nitrite concentration has a cGMP/PKG-dependent negative inotropic and muscular tone effect, characterized by a decrease in left ventricular relaxation and BP. This suggests that NRB may play an essential role in cardiac negative myodynamia and muscular tension.

In addition, studies have shown that the mechanisms of coronary dysfunction mainly involve inflammation, extensive and microvascular spasms, abnormal clotting and endothelial dysfunction [174, 175]. Endothelial dysfunction is a primary causative mechanism in patients with coronary heart disease risk factors. Endothelial dysfunction breaks down the endothelial l-NOS–NO pathway, contributing to a diminution in NO production, which leads to a decrease in endothelium-dependent diastolic effects and an increased risk of developing coronary artery disease, suggesting a critical function of NO in the regulation of endothelial function. Kanno et al. [176] similarly indicated that NO is vital in regulating cardiac function. They noted that in a healthy heart, NO produced by the endogenous NOS pathway reduces basal muscle force and plays a crucial role in safeguarding the myocardium from systolic/diastolic dysfunction, remodeling, and arrhythmias in the failing heart. Exogenous nitrate can produce NO through the NO_3_^−^/NO_2_^−^/NO pathway under the action of NRB, which is an independent alternative to the imbalance of endogenous pathways in a pathological state. Mechanisms by which NO acts include: (1) activation of soluble guanylate cyclase to produce cyclic guanosine monophosphate (cGMP) and relax vascular smooth muscle [177]; (2) increasing platelet cGMP levels to enhance anti-platelet agglutination and antithrombotic effects [178]; (3) decreases venous return and left ventricular end-diastolic pressure to diminish myocardial oxygen demand while increasing blood flow to the subendocardial; and (4) dilates coronary stenosis and increases collateral blood flow to directly increase myocardial oxygen supply [149, 179]. It is indicated that nitrate induces vasodilation of vascular smooth muscle through the NO_3_^−^/NO_2_^−^/NO pathway under the action of NRB, decreasing cardiac load and thus reducing left ventricular wall tension and end-diastolic pressure. It is a vital process in patients with stable ischemic heart disease (SIHD) as it not only reduces the load on the heart but also decreases the oxygen demand of the myocardium [180]. One study showed that nitrate could reduce myocardial ischemia and ischemic pain and increase exercise tolerance in stable and unstable angina pectoris. After acute myocardial infarction, nitrate can reduce ventricular dilatation, reducing pulmonary congestion and mitral regurgitation [181]. Besides, nitrate can also be used prophylactically before exercise, as they may improve exercise tolerance and avoid exercise-induced angina attacks [182, 183]. There is no doubt that nitrate have tremendous therapeutic potential in common cardiac diseases, such as angina pectoris. Currently, nitrate distributes coronary blood flow to ischemic areas through the NO_3_^−^/NO_2_^−^/NO pathway, thus improving local tissue hypoxia caused by illness as one of the many mechanisms to increase coronary perfusion. NRB, as the critical bacteria of nitrate metabolism to produce NO, few studies have clarified the potential mechanism of NRB in the recovery of coronary artery blood flow and reperfusion. The non-enzymatic and non-oxygen-dependent entero-salivary pathway is the primary mode of NO production, but an antibacterial mouthwash influences the effects produced by this pathway. A patient with angina pectoris reported in a case that had relief symptoms after discontinuation of antibacterial antiseptic mouthwash [184]. Therefore, we estimate that the NO_3_^−^/NO_2_^−^/NO path depends on the action of NRB to restore NO homeostasis and thus improve coronary perfusion.

Heart failure with reduced ejection fraction (HFrEF) is a fatal and disabling disease that is a significant public health concern. This disease is thought to be partially associated with the poor bioavailability of NO [181]. DN performs an essential part in treating conditions, including HFrEF, as a new source of human NO through intestinal salivary circulation and under the action of NRB. Some investigators have directly studied myocardial tissue from patients with HfrEF and found reduced cGMP levels, protein kinase G activity, and nitrite concentrations [185, 186]. Recent studies have shown that the impairment of coronary microvascular structure and function in HFpEF is primarily associated with decreased NO–cGMP bioavailability. When the organism is in a disease state, it leads to local microenvironmental hypoxia, which reduces endogenous NO production. We need new NO source pathways to restore NO homeostasis in the body [187–189]. The NO_3_^−^/NO_2_^−^/NO path is another strategy to enhance the NO–cGMP signaling pathway. Thus, NRB may serve as a suitable means to improve the efficiency of cardiac and peripheral NO signaling production at the early stages of the disease, thereby reducing the risk of disease progression to advanced settings. In addition, it has been shown that nitrite improves skeletal muscle mitochondrial efficiency, insulin sensitivity, and glucose uptake [190–192], so acute administration of inorganic nitrate treatment may enhance muscle strength in patients with HfrEF [192]. NRB is expected to be a probiotic to improve exercise capacity in patients with chronic HFpEF.

### Endocrine system

#### II diabetes mellitus and metabolic syndrome

Type 2 diabetes (T2D) and metabolic syndrome (MS) are chronic non-communicable diseases with high prevalence and rapid growth rates worldwide [193, 194]. MS is like T2D in clinical signs and is also predominantly insulin resistant. Patients with MS may also have T2D, a major manifestation of disorders of the body’s metabolism of protein, fat, carbohydrate, and other substances [195]. A WHO report predicts that by 2040, the number of adults with T2D will exceed 650 million worldwide [15]. These diseases require long-term control and treatment, are costly to treat, and are correlated with an increased risk of death. An unhealthy diet (e.g., high in fat, sugar, etc.) is important in the increased incidence of T2D and MS, which are often associated with oxidative stress, impaired NO signaling, and CD [196, 197]. Oral microflora disorder is also considered to be related to the occurrence and development of T2D and MS [78, 198–200]. Among them, NRB, which have a significant part in the metabolic homeostasis of oral microorganisms, are of interest, because they are involved in a major part of NO production (NO_3_^−^/NO_2_^−^/NO pathway) [201], supplying an alternative systemic source of NO. NO is considered to be a signal molecule closely related to carbohydrate metabolism [202], so the imbalance of NRB in oral microorganisms is a risk factor for the impairment of carbohydrate metabolism and the occurrence and development of T2D and MS [78]. Under hyperglycemia, the impairment of the l-NOS–NO pathway leads to the decrease of NO synthesis and bioavailability. Some studies have shown that increased blood glucose and advanced glycation end products (AGEs) can down-regulate the expression/activity [203] of eNOS through inflammation [204], and redox pathway [205].T2D can lead to elevated cytokine levels (e.g., TNF-α) and further downregulate eNOS expression [206]. This all leads to reduced endogenous NO synthesis and reduced bioavailability. In contrast, DN supplementation can compensate for the disturbance of the impaired enzyme-dependent pathway by promoting the NRB-dependent dietary NO_3_^−^/NO_2_^−^/NO pathway. Huang et al. [207] showed that mice deficient in eNOS evolve an MS-like phenotype with age. Carlstrom et al. [208] further showed that compared to controls, long-term dietary supplementation of nitrate decreased visceral fat accumulation, body weight gain, circulating triglycerides, and glycated hemoglobin (HbA1c) levels in mice and reversed the MS profile and prodromal diabetic phenotype in eNOS-deficient mice compared to controls. To investigate the relationship between T2D and MS and nitrate metabolism, Ohtake and his colleagues [209] found in a group of postmenopausal MS mouse models evoked by ovariectomy and a high-fat diet that these mice had lower circulating nitrate and nitrite levels compared to the corresponding controls and developed obesity, visceral adipocyte hypertrophy, and insulin resistance (IR), which would be avoided by nitrite treatment. Nyström and others [210] further pointed out that nitrite has dual stimulating effects on islet function, including indirect enhancement (increasing islet blood flow and redistribution through microcirculation) and direct insulin-promoting effect on β-cells. The insulin-promoting effect of nitrite is cGMP-dependent and involves the formation of active nitrogen and oxygen. In contrast, most nitrite in humans are produced by the reduction of DN by NRB. In addition, Khalifi et al. [211] investigated the effect of DN on glucose tolerance and lipids in a rat model of T2D induced by streptozotocin and nicotinamide injections. The results revealed that plasma nitrate and nitrite content decreased in these mice but recovered after nitrate supplementation, reducing hyperglycemia, and increasing blood lipid and glucose tolerance. Gheibi et al. [212] similarly found that obese T2D rats showed improved glucose tolerance, IR, and dyslipidemia after 2 months of DN supplementation compared to controls. These beneficial effects were correlated with increased GLUT4 expression in insulin-sensitive tissues and reduced gluconeogenesis, inflammation, and oxidative stress. In addition, Li et al. [213] found that DN supplementation attenuated the elevation of circulating triglycerides, total cholesterol, low-density lipoprotein (LDL) cholesterol, and high-density lipoprotein (HDL) cholesterol induced by dietary interventions (high-fat and high-fructose diets). In summary, inorganic nitrate/nitrite supplementation showed good therapeutic effects in animal models of T2D and MS, as widely reported in other literature as [39, 213–215]. These anions increased insulin secretion from beta cells [210, 216] and improved peripheral glucose utilization [217, 218], visceral fat accumulation, and circulating levels of triglycerides. We hypothesize that endogenous NO deficiency may be responsible for MS and T2D and that NRB performs a critical part in the alternative pathway (NO_3_^−^/NO_2_^−^/NO pathway). Joshipua et al. [219] studied the effects of oral disinfectant rinses on more than 900 people over 3 years. The participants who used the mouthwash regularly were found to have a 55% higher risk of developing prediabetes/diabetes than those who used it infrequently. The authors did not examine the underlying mechanism of this process, but it may be that the use of antiseptic mouthwash blocked the action of NRB. In human clinical trials, nitrate and nitrite were ineffective in improving metabolic disorders [220–222]. Gilchrist and his colleagues [223] found that while dietary supplementation with nitrate caused a noticeable rise in circulating nitrite in patients with T2D, it did not improve islet function in patients with T2D. The cause of the paucity of effect was thought to be linked to the fact that T2D patients were being treated with bisphosphonates. The mechanisms of action of inorganic nitrate and biguanides share some striking similarities in many respects [224], leading to no significant effect of DN supplementation in patients already receiving biguanides. Among these, the interaction of nitrate and biguanides with the host microbiota may be central to the underlying mechanism [225–227]. Studies have shown that the high relative abundance of NRB in human OC is associated with insulin resistance and reduced risk of prediabetes [78]. Both animal and human trials have shown that alterations in the oral microbiota of patients with T2D lead to reduced nitrate reduction in the OC, decreased NO bioavailability, and the development of IR. In contrast, inorganic nitrate can modulate the oral microbiota by raising the number of health-related NRB and reducing the abundance of *Prevotella* and *Weyongococcus* spp., thereby increasing NO production and improving NO utilization. In addition, dietary epidemiological research has revealed that increased intake of nitrate-rich vegetables can convey weight loss and anti-diabetic effects and prevent the development of T2D [228, 229]. A review has discussed in detail how inorganic nitrate can improve the oral microbial community of patients with T2D and make it plays a probiotic role [230]. Further understanding of the pathophysiological mechanisms of T2D and MS will help develop new preventive and therapeutic strategies. Future animal studies or clinical trials will assess the potential beneficial effects of dietary supplementation with NRB in patients with metabolic diseases, including T2D. Combining the effects of the interaction of NRB with nitrate, restoring the oral microbiota of patients with T2D or MS to a state comparable to health is a state-of-the-art approach [41], which could enhance systemic NO production and offer an alternative way in the presence of impaired enzyme-dependent endogenous pathways. These findings may offer innovative nutritional prevention and treatment strategies for T2D and MS.

### Nonalcoholic fatty liver

Non-alcoholic fatty liver disease (NAFLD) is the most common liver disease worldwide, called hepatic steatosis or fatty liver, and is closely related to overweight, obesity, and MS [231, 232]. NAFLD can be reversed with weight loss and movement, which can also develop into severe diseases, including non-alcoholic steatohepatitis, fibrosis, and liver cirrhosis [233, 234]. Recent studies have shown that steatosis can be avoided by simple dietary approaches in rodent and human models of MS [209, 235]. In the latest trial, DN was proven to modify vascular function in patients with hypercholesterolemia [151]. Sonoda et al. [16] further found that high nitrate levels in diet positively affect liver steatosis associated with metabolic syndrome. DN provides fuel for the NO_3_^−^/NO_2_^−^/NO pathway, which can reverse many characteristics of metabolic syndrome and liver steatosis in high-fat diet mice (whether combined with NO synthase inhibitor (l-NAME) or not) [6, 236, 237]. Many scholars believe impaired NO bioavailability and signaling may be a candidate mechanism for hepatic steatosis [28, 238]. Lázár et al. [239] noted that the beneficial effects of nitrate metabolism could be attributed to its reduction of nitrite followed by further generation of NO species and activation of soluble guanylate cyclase [114, 240]. Furthermore, Cordero-Herrera et al.’s [15] in vivo disease model and in vitro studies using HepG2 cells and primary human hepatocyte spheres show that stimulating the NO_3_^−^/NO_2_^−^/NO pathway may slow down the evolution of hepatic steatosis by activating AMP-activated protein kinase (AMPK) signal transduction and reducing NOX-derived oxidative stress. These beneficial effects of nitrate were not present in germ-free mice, suggesting a central part of the host microbiota in the bioactivation of nitrate, which is necessary for DN to avoid steatosis. These findings may have implications for developing new strategies for the prevention and treatment of hepatic steatosis associated with metabolic disorders based on NRB.

### Neuro system

Sympathetic excitation is accompanied by the increase of AngII signal and oxidative stress and impairs the bioavailability of NO [18, 19]. DN supplementation may influence the body's systemic health by modulating sympathetic activity and thus reducing vascular tone, regulating BP, and altering mental behaviors. These beneficial impacts of DN may be mediated through the inhibitory effect of the NO_3_^−^/NO_2_^−^/NO pathway on sympathetic excitation, in which NRB play an important role [18, 241]. Guimarães et al. [18] first demonstrated that long-term nitrate supplementation inhibited or restored regular sympathetic activity in an animal model of angiotensin-converting enzyme II-induced hypertension. However, this experiment did not directly verify the role of NRB but instead emphasized the significance of NRB in the regulation of NO homeostasis and signaling via the NO_3_^−^/NO_2_^−^/NO pathway. NO has a significant function in regulating synaptogenesis and neurotransmission in the central and peripheral nervous system [242]. There is evidence that basal NO production and NO bioavailability are reduced in diabetic patients [17]. Oghbaei et al. [243] used a ureazotocin-induced diabetic male rat model to demonstrate that long-term supplementation with DN can affect testicular function and structure in these rats through the hypothalamic–pituitary–gonadal axis, thereby improving fertility parameters. Subsequently, Keyhanmanesh et al. [244] also demonstrated the beneficial therapeutic effect of DN supplementation on testicular damage in streptozotocin-induced diabetic male rats. These findings are correlated with the increase in miR-34b and decrease in p53 mRNA, of which the role of NRB has not been revealed. We speculate that NRB may function as an essential part of this process. In addition, García-Jaramillo et al. [245] pointed out that DN supplementation altered the metabolomic profile of the zebrafish brain and led to mild anxiety-like behaviors. They suggest that DN supplementation can deplete brain metabolites (e.g., reduction of γ-aminobutyric acid and its precursor glutamine) by modulating neural activity, a process in which NO plays a vital role. We hypothesize that NRB is closely related to the operation of regulation of neural activity in humans, pending further experimental evidence. In addition to regulating nerve activity, DN can improve cognitive function by regulating blood flow to the brain and reducing response times in neuropsychological tests [246, 247]. Vanhatalo et al. [13] found that after DN supplementation, the microbiome module associated with pro-inflammatory metabolism decreased, and the microbial module with NRA increased and suggested that this microbial module alteration may be related to improvements in age-induced cognitive impairment. The relationship between NRB and cognitive function still needs further experimental validation.

### Respiratory system

#### Bronchopulmonary dysplasia

Bronchopulmonary dysplasia (BPD) is the most common severe respiratory complication in preterm infants and contributes significantly to the mortality of preterm infants [22, 248]. Currently, we lack treatments to block the progression of the disease as well as biomarkers to predict BPD. Despite the complexity of the aetiology and pathogenesis of BPD (which remains to be elucidated), recent studies demonstrated a relationship between the microecological environment of the digestive system (including the oral, respiratory, and intestinal tracts) and BPD. Among them, the correlation microbiome mediates NRA that regulates NO bioavailability and signaling, and defective NO bioavailability can lead to an increased incidence of BPD. Therefore, the bacteria with NRA in the OC may be the key to predicting and treating BPD [77, 249, 250]. In a single-center prospective cohort study, Gentle et al. [251] found that NRA peaked at 29 weeks of gestational age; when infants were categorized and compared by whether they had BPD, those with BPD had noticeably lower NRA at 29 weeks of gestational age than those without BPD. High NRA was associated with a lower incidence of BPD. This suggests that oral microflora and NRA may play a role in the occurrence of BPD in very premature infants. The difference in NRA mediated by oral microflora may provide a non-invasive biomarker for the development of BPD and has the potential for targeted therapy. In addition, studies have shown that significant changes in respiratory tract microecology (such as abnormal microbial diversity and differences in evolutionary patterns) have taken place in premature infants before the occurrence of BPD [252, 253]. Similarly, Wagner et al. [254] noted that the respiratory tract of infants with severe BPD has a higher weekly detectable microbial load than that of infants with mild BPD (with a lower colonization rate of Staphylococcus in the respiratory tract of infants with severe BPD). This suggests that the colonization pattern of respiratory microflora in premature infants may be a marker for predicting the severity of BPD. A review has elucidated in some detail the relationship between respiratory microbes and BPD and their primary mechanisms of action in the pathogenesis of BPD, suggesting that intervention of respiratory microecology with probiotics holds promise to become a new policy in the therapy of BPD [22]. Furthermore, there is growing evidence that the respiratory and gut microecological environments can interact, affecting the development of BPD. Dysregulation of the respiratory and gut microbiota can cause immune disorders and exacerbate the consequences of disease by stimulating inflammatory processes [255, 256]. Therefore, maintaining respiratory and intestinal microecological balance can significantly improve BPD. Studies have shown that there are also bacteria with NRA in the respiratory tract and intestinal tract. These bacteria are essential in regulating the microecological balance of the respiratory tract and intestinal tract [257, 258]. Therefore, we hypothesize that NRB may be highly promising targets for predicting and treating BPD. At present, NO and NO-producing precursors have been evaluated as potential BPD predictive markers and prevention methods, but the relationship between nitrate-reducing bacteria and BPD and the role of nitrate-reducing bacteria in predicting and treating BPD need to be further studied.

### Urology systems

#### Chronic kidney disease

Chronic kidney disease (CKD) is a primary health problem affecting 8–16% of the global population [259]. Studies have shown that the microflora of patients with CKD is impaired (biological imbalance), increasing the number of potentially pathogenic and pro-inflammatory bacteria that can produce uremic toxins that facilitate the progression of CKD and decrease the production of enzymatic NO [24]. It has been confirmed in most animal experiments that the bioavailability of NO in patients with CKD is reduced. Since the decreased bioavailability of NO and increased oxidative stress are the key to the occurrence and development of renal disease [260], the therapeutic targets for NO may be beneficial [261–263]. In several disease models, DN supplementation has increased NO production and reduced endothelial dysfunction [145, 264]. In addition, supplementation with nitrite has been demonstrated to prevent or minimize renal damage directly by increasing NO production but has not been shown to improve the prognosis of patients with CKD [265, 266]. Similarly, we can also prevent or reduce renal impairment by increasing NO production through DN supplementation. Different ways of stimulating the NO_3_^−^/NO_2_^−^/NO pathway have been presented to avoid renal ischemia–reperfusion damage. Nitrate supplementation can improve renal injury in ischemia–reperfusion animal model [267, 268]. Two weeks of DN supplementation (1 mmol/kg/day) enhanced glomerular perfusion and filtration, prevented a decrease in glomerular filtration rate and attenuated glomerular and tubular injury in mice 2 weeks after ischemic renal injury [269]. Mechanistically, these renal protective effects associated with nitrate supplementation have been linked to reduced oxidative stress and increased NO bioavailability. A review detailed that the enhanced NO_3_^−^/NO_2_^−^/NO pathway may perform an antioxidant role through different targets and cellular mechanisms (including regulation of mitochondrial function, reduction of ROS produced by NOX and XO, and restoration of eNOS function) [25]. Although NRB is the only human symbiotic bacteria in the NO_3_^−^/NO_2_^−^/NO pathway, few studies clarify the relationship between NRB and CKD. As the impact of microorganisms on human health is gradually revealed, the microbiota can regulate the nitrogen cycle in various ways (e.g., through nitrification and denitrification, regulation of reduction and oxidation reactions, and through biological and chemical reactions) and play a crucial role in NO production [21, 270, 271]. One review has elaborated on how microorganisms regulate the nitrogen cycle [24]. Therefore, the effect of NRB on CKD may be a topic worth exploring in depth. In conclusion, the nitrogen cycle is an essential component of CKD prevention. Improper regulation of any part of the nitrogen cycle may promote the development of CKD. Regulating the nitrogen cycle by developing interventions that target NO production (e.g., supplementing NRB to regulate the microbiota) and thereby increasing NO production has the potential to reduce the risk of CKD. So far, few studies have fully described the role of NRB in the oral, esophageal or gastrointestinal microflora of patients with CKD. Therefore, we should focus on exploring this aspect in future studies and experiments, which may provide promising innovative ideas for predicting and treating CKD.

## Summary and discussion

NRB perform a vital part in human health and diseases, because human beings lack the enzyme mechanism of reducing nitrate to nitrite, so we can only rely on NRB to reduce nitrate and make the NO_3_^−^/NO_2_^−^/NO pathway play a substitute role in the inactivation of the classical NOS pathway, thus restoring the balance of NO. In addition, NRB function as a critical regulator in the human floras. NRB have many beneficial effects on the body by restoring NO balance through the NO_3_^−^/NO_2_^−^/NO pathway or by regulating floras balance, including improving the hypofunction of salivary glands due to radiotherapy for HNT, regulating oral floras, protecting the stomach by improving gastric MBF and gastric mucus production, regulating intestinal floras, improving hypertension and pulmonary hypertension, alleviating heart disease, diabetes, MS, fatty liver, bronchopulmonary dysplasia, and chronic kidney disease. This review comprehensively summarizes the potential relationship between NRB and human health and conditions, an aspect that has received little attention and has not been summarized. NRB may be the key to the production of NO by nitrate metabolism and affect human health and may also be important bacteria for human health regulation. Our follow-up study can use these bacteria as a breakthrough to study the potential mechanism between nitrate metabolism, health, and disease. Therefore, this article accumulates a theoretical foundation for further studies on the effects of NRB on human health and diseases and stimulates more thoughts. The relationship between the function of microorganisms and the balance of human health and disease is becoming increasingly clear. Among them, studying the effects of bacteria with nitrate reductase on human systemic health and disease is still at an early stage. With the accelerated evolution of high-throughput sequencing and bioinformatics technologies, comprehensive microbiome characterization of NRB in different disease populations has become possible. According to the current research, the related studies on the relationship between NRB and human health and diseases are based on digestive tract floras (including the oral cavity, esophagus and gastrointestinal tract). This suggests a close relationship between NRB in our digestive tract and human health, which is worthy of in-depth excavation. Furthermore, NRB can influence the health of other body organs through the oral–intestinal axis, which can provide innovative ideas for disease diagnosis and treatment, such as finding biomarkers for specific diagnoses of diseases and treating-related conditions through probiotics by screening the changes of NRB in the digestive tract. In addition, the beneficial effect of NRB on the human body may be a protective mechanism of disease state or ageing, which not only indicates that NRB may be a potential therapeutic target but also may be a beneficial flora for improving age-related diseases, which needs to be further confirmed by more clinical trials. Recently, the modulation of the gut microbiota by NRB as beneficial commensals has attracted attention because of the potential effect of these floras on a variety of conditions and also as supplements to improve exercise performance [272, 273]. Using diet or supplements to change the microflora to increase the conversion of nitrate to nitrite, to increase the production and utilization of NO signal, which opens a new way in the field of sports performance. The use of high-throughput sequencing technology and biological information technology to understand the bacteria with nitrate reductase activity has been deepened, and a large number of data and information have been obtained. How to effectively transform big biological data into a clinical diagnosis and treatment method with practical application value and then provide effective personalized medical services for patients, there are still many problems to be solved. The growing development of metagenomics and high-throughput sequencing technologies significantly extended the human knowledge of the association between NRB and systemic diseases. Understanding the specific mechanism of NRB affecting human health and diseases and controlling NO_3_^−^/NO_2_^−^/NO metabolism is of great significance for preventing and treating human systemic diseases.

## Data Availability

This review does not involve statistics, and all the pictures are original.
